# Mining Chemotherapy Resistance Related Genes in Breast Cancer to Construct a New Prognosis Prediction Model‐Based on GEO Database and Real‐World Study

**DOI:** 10.1155/humu/1378458

**Published:** 2026-07-29

**Authors:** Zhaozhen Qiu, Xiaodong Dai, Jianfeng Zeng

**Affiliations:** ^1^ Department of Breast Surgery, The Second Affiliated Hospital of Fujian Medical University, Quanzhou, Fujian, China, fjmu.edu.cn; ^2^ Department of Intensive Care Unit, Quanzhou First Hospital, Quanzhou, Fujian, China

**Keywords:** breast cancer, drug resistance gene, prognosis prediction model

## Abstract

**Objective:**

The chemotherapy resistance genes in breast cancer are closely related to prognosis. This study is aimed at exploring the key genes that may be involved in chemotherapy resistance of breast cancer and establishing a prognostic model.

**Methods:**

Using data from the GEO database, differentially expressed genes (DEGs) related to chemotherapy resistance in breast cancer were identified. Univariate and multivariate Cox regression were used to identify the association between DEGs and prognosis. Subsequently, functional analysis was conducted to characterize the functions of DEGs. In addition, immune‐related analysis was performed to study the functions of these hub genes. LASSO‐Cox regression analysis narrowed the range of hub genes. A DRFS prognostic nomogram model was constructed using the hub genes. A total of 60 breast cancer patients from the Second Affiliated Hospital of Fujian Medical University were selected as the external validation set.

**Results:**

By comparing the gene expression profiles of the Rx_Insensitive group and the Rx_Sensitive group, 162 DEGs were screened out, among which 53 DEGs were upregulated and 109 DEGs were downregulated. Univariate Cox regression analysis of the 162 DEGs with survival showed that GREB1, DACH1, STAP1, TDRD12, and SCGB1D2 were significantly associated with prognosis (all *p* < 0.05). Further multivariate Cox regression analysis revealed that GREB1 (HR = 0.653), DACH1 (HR = 1.217), STAP1 (HR = 1.140), and SCGB1D2 (HR = 1.074) were independent risk factors for prognosis (all *p* < 0.05). Moreover, the expression levels of GREB1, DACH1, STAP1, and SCGB1D2 were significantly correlated with the infiltration levels of various immune cells (*p* < 0.05). Based on these five breast cancer chemotherapy resistance‐related genes, a new prognostic model for breast cancer was constructed. The 1‐year AUC of this model was 0.748, 3‐year AUC was 0.735, and 5‐year AUC was 0.679. In the validation set, the 1‐year AUC was 0.744, 3‐year AUC was 0.696, and 5‐year AUC was 0.650. The calibration curve showed that the predicted probabilities of the model were close to the true values. The model′s prediction accuracy on the external validation set for 1 year was 0.823.

**Conclusions:**

The prognostic model developed based on the five breast cancer chemotherapy resistance‐related genes (GREB1, DACH1, STAP1, TDRD12, and SCGB1D2) has good predictive performance for BRCA patients.

## 1. Introduction

Breast cancer is one of the most common malignant tumors among women worldwide, with its incidence and mortality rates remaining high, seriously threatening the life and health of women. Neoadjuvant chemotherapy (NAC) has become an important component of the internationally recognized standard treatment for breast cancer. For patients with locally advanced disease and a strong desire for surgery, NAC can significantly improve the success rate of surgery and the rate of tumor resection; for patients with high requirements for breast preservation, it can also provide new solutions [1]. If the NAC regimen is effective, it has important guiding value when designing further treatment plans after surgery. Throughout the entire treatment process of breast cancer patients, NAC plays an increasingly important role, and this has been widely recognized internationally. Randomized clinical trials have shown that patients who achieved pathological complete response with NAC have better prognosis than those who did not [2], making it possible for NAC to quickly determine the response to treatment and the chemotherapy sensitivity in the body [3]. In breast cancer NAC, anthracyclines and taxanes are the most commonly used chemotherapy drugs, and chemotherapy regimens based on anthracyclines and taxanes can reduce the mortality rate of breast cancer by approximately one‐third [4]. Although significant progress has been made in the diagnosis and treatment of breast cancer in recent years, chemotherapy resistance remains one of the key factors limiting the therapeutic effect of breast cancer [5]. Chemotherapy resistance refers to the gradual development of resistance of tumor cells to chemotherapy drugs under the action of these drugs, resulting in the inability of chemotherapy drugs to effectively kill tumor cells and allowing the tumor to continue to grow and spread. This not only reduces the survival rate of patients but also increases the difficulty and cost of treatment.

Currently, the prognosis assessment of breast cancer mainly relies on clinical and pathological features, such as tumor size, lymph node metastasis status, and histological grade. However, these traditional indicators have certain limitations in predicting patient prognosis and cannot accurately reflect the biological behavior and individual differences of the tumor. In recent years, with the rapid development of genomics technology, more and more breast cancer chemotherapy resistance‐related genes have been discovered and identified [6, 7]. These genes play an important role in the biological processes of tumor cell proliferation, apoptosis, invasion, and resistance. Through in‐depth research on these genes, it is expected to construct a more precise breast cancer prognosis model, providing more powerful support for clinical treatment decisions.

Constructing a prognostic model based on genes related to chemotherapy resistance in breast cancer can not only help doctors make more accurate prognostic assessments for patients, but also provide a basis for the formulation of individualized treatment plans. By screening out genes closely related to chemotherapy resistance, it is possible to predict the sensitivity of patients to chemotherapy drugs in advance, thereby avoiding unnecessary chemotherapy and side effects. Moreover, this model can also provide new directions for basic research on breast cancer, helping to deeply understand the molecular mechanism of chemotherapy resistance in breast cancer and providing a theoretical basis for the development of new therapeutic targets and drugs. In summary, this study is aimed at constructing a new prognostic model based on genes related to chemotherapy resistance in breast cancer, with the expectation of improving the treatment effect and survival rate of breast cancer patients and improving their prognosis.

## 2. Materials and Methods

### 2.1. Data Sources and Preprocessing

The dataset GSE25066 was downloaded from the GEO database (https://www.ncbi.nlm.nih.gov/geo/browse/?view=series). As mentioned earlier [8], the general clinical data of the patients were obtained. This dataset is the chip expression profile data (Expression profiling by array) of the response of breast cancer patients to neoadjuvant paclitaxel‐anthracycline chemotherapy. It contains 339 samples with Rx_Insensitive and 169 samples with Rx_Sensitive. The dataset is from a prospective multicenter study from June 2000 to March 2010 [8]. After downloading the Series Matrix File(s) and the GPL96 platform files of the GSE25066 dataset, the probe expression matrix was converted into a gene expression matrix using the R software. Probes without corresponding ones were removed. For multiple probes corresponding to the same gene, their expression values were summed and averaged.

### 2.2. Identification of Differentially Expressed Genes (DEGs)

R software was used to verify whether the chip data has been log2‐standardized. The “limma” R package was utilized to conduct the analysis of gene differential expression. The principle is to establish a linear model between the control group and the disease group, and then evaluate the differential expression and gene set test through empirical Bayesian adjustment. DEGs were identified using *p* < 0.05 and |log2FC| > 0.5 as thresholds. The R package “pheatmap” was used to draw the expression gene heatmap.

### 2.3. Univariate and Multivariate Cox Regression Analysis

Through univariate Cox regression analysis, DEGs related to prognosis were screened. Then, multivariate Cox regression analysis was conducted to determine which prognostic factors independently predicted patient survival. A *p* value < 0.05 was considered statistically significant when using the “survival” package. Kaplan–Meier analysis was performed to compare the distant recurrence‐free survival (DRFS) of patients with high expression and those with low expression. Patients with expression values below the 25th percentile were defined as the low‐expression group, those between the 25th and 75th percentiles as the medium‐expression group, and those with expression values above the 75th percentile as the high‐expression group. Logrank test was used to examine the differences.

### 2.4. Function Analysis

The functions of DEGs were analyzed through the DAVID database (https://david.ncifcrf.gov/), including Gene Ontology (GO) and Kyoto Encyclopedia of Genes and Genomes (KEGG).

### 2.5. Functional Analysis of Hub Genes Related to Immunity

TISIDB is a portal website for the interaction between tumors and the immune system, and it is also a database for tumor immune analysis. To extract and quantify the immune infiltration expression of the gene expression matrix of Rx_Insensitive and Sensitive samples, the gene sets of immune cells were downloaded from the TISIDB database (http://cis.hku.hk/TISIDB/download.php), including 28 immune cell gene sets. The single‐sample gene set enrichment analysis (ssGSEA) was used to determine the immune infiltration level of the corresponding datasets. ssGSEA is a tool for immune infiltration analysis, which can estimate the degree of immune infiltration in each sample. The principle of ssGSEA indicates that, for the gene expression matrix of disease samples, ssGSEA first sorts the expression values of each gene in each sample, obtaining the rank of each gene among all genes, and all genes are regarded as background genes. Then, for the input immune cell gene sets, genes existing in the background genes are found from the immune cell gene sets and counted, and the expression levels of these genes are summed. Subsequently, based on the above evaluation, the expression level of any gene (the sum of the expression levels of the background genes and the genes in the immune cell gene set) is calculated to obtain the enrichment score of each gene in the immune cells. Then, the enrichment scores are recalculated in random order, and the calculation is repeated 1000 times to obtain 1000 enrichment scores. The *p* value is calculated based on the distribution of the gene enrichment scores. Subsequently, the enrichment scores and *p* values of each gene included in the immune cells are calculated. The “GSVA” R package is used to score the transformed gene expression matrix by ssGSEA, and the “pheatmap” R package is used to draw the immune infiltration heatmap.

In order to compare the differences in immune infiltration between the Rx_Insensitive group and the Sensitive group, the sample grouping situation and the ssGSEA expression matrix results were integrated. The abundance of immune cells in the two groups was compared using the “ggpubr” R package, and a rank sum test was conducted. A boxplot comparing the immune infiltration scores of the Rx_Insensitive group and the Sensitive group was drawn.

To analyze the correlation between DEGs and immune infiltration, the “psych” package of R software was used to analyze the ssGSEA expression matrix and the intersection difference gene expression matrix. The correlation analysis method was Spearman correlation, and a correlation heatmap was constructed. A correlation was considered significant if the *p* value was less than 0.05.

### 2.6. LASSO Regression Analysis

The LASSO regression analysis was conducted using the “glmnet” package of R software. The optimal regularization parameter was selected through cross‐validation to obtain a more robust model.

### 2.7. Construction of Prognostic Model

In order to analyze the predictive effect of genes related to chemotherapy resistance in breast cancer on the prognosis of breast cancer patients, the prognosis gene expression matrix and sample grouping information were used as input files. A logistic regression model was constructed using the “rms” program package of R software. A nomogram risk prediction chart was built to calculate the quartile values of each gene. Expression values below the P25 percentile were classified as low expression, values between the P25 and P75 percentiles were classified as moderate expression, and values above the P75 percentile were classified as high expression. Thus, the risk scores of each hub gene were calculated, and the calibration curve and receiver operator characteristic (ROC) curve were used for verification to evaluate the identification ability of the nomogram model.

### 2.8. External Data Set Validation

From January 2025 to March 2025, 60 breast cancer patients admitted to the Second Affiliated Hospital of Fujian Medical University were collected. All patients underwent NAC with paclitaxel and anthracycline drugs. After completing NAC, all patients underwent surgical treatment at our hospital, including modified radical mastectomy, breast‐conserving surgery, and simple breast resection. The study involving the external cohort of 60 breast cancer patients was approved by the Institutional Ethics Committee of the Second Affiliated Hospital of Fujian Medical University. Written informed consent was obtained from all participants prior to sample collection and data analysis.

The inclusion criteria are as follows: (1) female patients aged ≥ 18 years at diagnosis; (2) diagnosed with invasive breast cancer; (3) treated with modified radical mastectomy, breast‐conserving surgery, or simple resection; (4) unilateral breast tumor without distant metastasis; (5) complete clinical data and pathological results before and after chemotherapy, and reliable; (6) no previous radiotherapy or chemotherapy. The exclusion criteria are as follows: (1) patients with M1 at initial diagnosis; (2) patients diagnosed with bilateral primary breast cancer; (3) patients with inflammatory or other atypical breast cancer; (4) patients who changed chemotherapy regimens during chemotherapy; (5) patients with concurrent other malignant tumors; (6) patients with incomplete clinical and pathological data or lost follow‐up.

RNA sequencing technology was used to detect the expression levels of GREB1, DACH1, signal transduction adaptor proteins 1 (STAP1), Tudor Domain‐Containing Protein 12 (TDRD12), and SCGB1D2 in the cancer tissues of the patients, and these 60 patients were followed up. The follow‐up period ended in April 2026. The main focus of this study was to evaluate the DRFS of the patients. The survival rate of the 60 patients was evaluated using a nomogram model, and the clinical predictive value of the model was evaluated using the ROC curve.

### 2.9. Statistical Processing

All data analyses were performed using R (Version 4.5.1). Independent prognostic survival factors were determined through univariate and multivariate Cox regression analysis. The predictive value of the model for DRFS was evaluated through Kaplan‐Meier analysis. The predictive value of the model for DRFS was assessed through Kaplan‐Meier analysis. Time‐dependent ROC curves for 1, 3, and 5 years of model prediction accuracy were evaluated. *p* < 0.05 was considered statistically significant. The *p* values are as follows: ns, not significant;  ^∗^ *p* < 0.05;  ^∗∗^ *p* < 0.01; *p* < 0.001.

## 3. Results

### 3.1. Differential Genes Related to Chemotherapy Resistance in Breast Cancer

**Figure 1:**
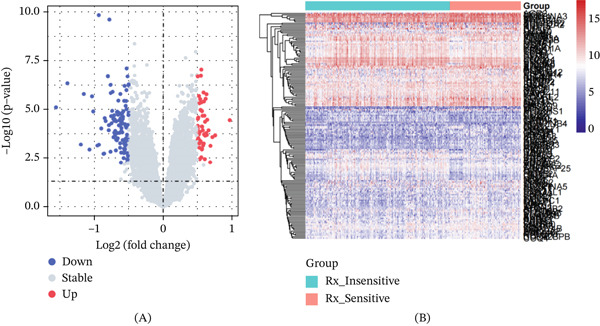
Screening of differentially expressed genes related to chemotherapy resistance in breast cancer. (A) Volcano plot of differentially expressed genes. (B) Heatmap of DEGs expression between Rx_Insensitive and Rx_Sensitive.

By comparing the gene expression profiles of the Rx_Insensitive group and the Rx_Sensitive group, we identified 162 DEGs. Among them, 53 DEGs showed increased expression, whereas 109 DEGs showed decreased expression. The results are shown in Figure 1A,B.

### 3.2. DEGs Related to Survival

**Figure 2:**
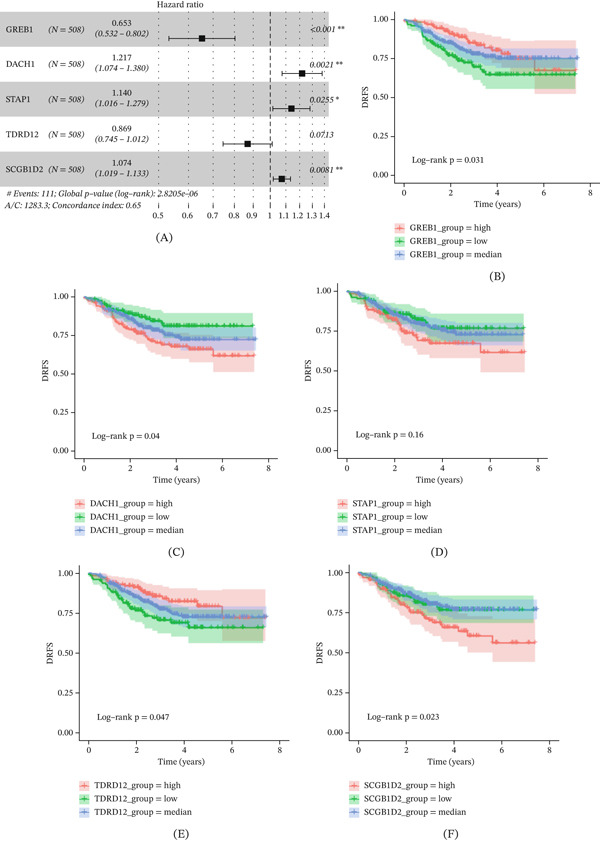
Survival analysis for screening of drug resistance DEGs related to survival: (A) multivariate COX regression analysis; (B) survival curve of GREB1; (C) survival curve of DACH1; (D) survival curve of STAP1; (E) survival curve of TDRD12; (F) survival curve of SCGB1D2.

Further analysis was conducted on the relationship between 162 DEGs and survival. Univariate Cox regression analysis revealed that GREB1, DACH1, STAP1, TDRD12, and SCGB1D2 were significantly associated with prognosis (all *p* < 0.05), whereas the remaining genes showed no statistically significant difference in prognosis (*p* > 0.05), as shown in Table 1. Multivariate Cox regression analysis indicated that GREB1 (hazard ratio [HR] = 0.653 [0.532–0.802]) is an independent protective factor (*p* < 0.05), and DACH1 (HR = 1.217 [1.074–1.380]), STAP1 (HR = 1.140 [1.016–1.279]), and SCGB1D2 (HR = 1.074 [1.019–1.133]) are independent risk factors for prognosis (all *p* < 0.05), with TDRD12 (HR = 0.869 [0.745–1.012]), *p* > 0.05, as shown in Figure 2A. KM curve analysis revealed that patients with high expression of GREB1 and TDRD12 had significantly better disease‐free survival (DFS) rates than those with low expression, whereas patients with low expression of DACH1 and SCGB1D2 had significantly better DFS rates than those with high expression, and the differences were statistically significant (*p* < 0.05), as shown in Figure 2B–F. The inconsistency between Cox regression and Kaplan–Meier analysis may be due to information loss caused by dichotomization/trichotomization of continuous variables. Given the different statistical assumptions and information utilization between Cox regression and Kaplan–Meier survival analysis, we integrated both approaches to comprehensively evaluate the prognostic relevance of the candidate genes.

**Table 1:** Univariate Cox regression analysis.

Gene	*p*	HR (95% CI for HR)	Gene	*p*	HR (95% CI for HR)
TJP3	0.376	0.948 (0.844–1.070)	SYT17	0.340	0.946 (0.844–1.060)
AREG	0.768	0.980 (0.854–1.120)	SCUBE2	0.783	1.010 (0.938–1.090)
RABEP1	0.193	1.130 (0.941–1.350)	CCR3	0.323	1.070 (0.934–1.230)
CD53	0.181	1.120 (0.950–1.310)	PGR	0.871	0.993 (0.913–1.080)
COTL1	0.933	0.993 (0.844–1.170)	KLRC1	0.836	1.010 (0.914–1.120)
CERS4	0.838	1.010 (0.903–1.130)	LYZ	0.503	1.040 (0.924–1.180)
ASTN2	0.590	0.954 (0.803–1.130)	SERPINA3	0.667	1.020 (0.928–1.120)
THEMIS2	0.723	0.968 (0.810–1.160)	FOXA1	0.384	1.050 (0.939–1.180)
TFF1	0.505	1.020 (0.960–1.090)	RAC2	0.828	1.010 (0.897–1.150)
FAM174B	0.250	1.110 (0.927–1.340)	SLC4A8	0.228	1.070 (0.959–1.190)
CITED1	0.776	0.983 (0.874–1.110)	PLEKHS1	0.849	0.991 (0.903–1.090)
**GREB1**	**0.046**	**0.840 (0.708–0.997)**	SLC16A6	0.848	1.010 (0.910–1.120)
IRX5	0.912	0.991 (0.848–1.160)	AZGP1	0.830	0.989 (0.896–1.090)
NUMA1	0.852	1.020 (0.866–1.190)	TRAT1	0.445	1.040 (0.937–1.160)
ELOVL2	0.641	0.973 (0.867–1.090)	RSAD2	0.259	1.080 (0.944–1.240)
EVI2B	0.416	1.060 (0.919–1.230)	CORO1A	0.420	1.050 (0.937–1.170)
SLC39A6	0.470	1.040 (0.933–1.160)	ARRB2	0.614	1.030 (0.910–1.170)
TOX3	0.789	1.010 (0.940–1.090)	GSTM3	0.726	1.020 (0.906–1.150)
XBP1	0.176	1.100 (0.958–1.260)	BIN2	0.574	1.030 (0.930–1.140)
LCK	0.700	0.974 (0.852–1.110)	IL32	0.264	0.952 (0.873–1.040)
S100A4	0.595	1.040 (0.889–1.230)	NPL	0.487	0.964 (0.870–1.070)
SERPINA5	0.977	0.999 (0.922–1.080)	GATA3	0.786	0.988 (0.903–1.080)
SRGN	0.275	1.070 (0.944–1.220)	CD52	0.227	1.060 (0.966–1.160)
CYTIP	0.138	1.120 (0.964–1.300)	PP14571	0.703	1.020 (0.931–1.110)
KRT19	0.693	0.976 (0.866–1.100)	DAK	0.169	0.919 (0.814–1.040)
IRS1	0.772	1.020 (0.885–1.180)	GSTT1	0.361	0.957 (0.870–1.050)
CD151	0.201	0.913 (0.795–1.050)	CTSW	0.988	1.000 (0.884–1.130)
ARHGAP25	0.192	1.090 (0.957–1.250)	C6orf211	0.646	1.030 (0.911–1.160)
CD3D	0.507	1.050 (0.910–1.210)	SIX1	0.354	1.060 (0.941–1.190)
TRAC	0.504	1.050 (0.907–1.220)	TBC1D9	0.255	1.070 (0.949–1.220)
ZNF446	0.400	0.941 (0.817–1.080)	APOD	0.870	0.992 (0.905–1.090)
AGR2	0.186	1.040 (0.983–1.090)	C1orf21	0.724	0.979 (0.867–1.100)
INHBB	0.928	0.993 (0.860–1.150)	BSCL2	0.401	0.955 (0.859–1.060)
CD69	0.948	1.000 (0.870–1.160)	GYS2	0.187	1.090 (0.960–1.230)
**DACH1**	**0.021**	**1.130 (1.020–1.250)**	STC2	0.807	0.985 (0.876–1.110)
LILRB2	0.434	0.947 (0.828–1.080)	WNT4	0.489	0.963 (0.864–1.070)
TFF3	0.735	1.010 (0.940–1.090)	DNAJC12	0.451	1.040 (0.945–1.140)
VAV3	0.940	1.000 (0.882–1.150)	LRMP	0.672	1.030 (0.913–1.150)
GRP	0.246	1.070 (0.956–1.190)	IFT74	0.134	0.919 (0.822–1.030)
MLPH	0.428	1.050 (0.935–1.170)	**STAP1**	**0.049**	**1.130 (1.000–1.270)**
MAPK3	0.808	0.982 (0.845–1.140)	**TDRD12**	**0.037**	**0.854 (0.736–0.991)**
ANO1	0.113	1.100 (0.977–1.250)	CCL8	0.742	0.980 (0.870–1.100)
CDC42BPB	0.078	0.898 (0.797–1.010)	SERPINB4	0.345	1.060 (0.941–1.190)
MNAT1	0.409	0.950 (0.843–1.070)	CLDN3	0.686	0.976 (0.868–1.100)
BARD1	0.332	0.944 (0.839–1.060)	TRPM4	0.496	0.966 (0.876–1.070)
ACADSB	0.198	1.080 (0.960–1.220)	GPR171	0.523	1.040 (0.926–1.160)
MYO6	0.391	0.939 (0.812–1.080)	ALDH3B2	0.749	1.020 (0.914–1.130)
IGFBP4	0.954	0.997 (0.897–1.110)	LAMP3	0.098	0.917 (0.827–1.020)
TRBC1	0.400	1.050 (0.933–1.190)	**SCGB1D2**	**0.020**	**1.060 (1.010–1.110)**
PTPRC	0.258	1.080 (0.947–1.230)	KRT15	0.608	1.020 (0.953–1.090)
EFHC1	0.911	1.010 (0.911–1.110)	TMC5	0.744	0.982 (0.880–1.100)
SIAH2	0.743	0.979 (0.863–1.110)	PIP	0.442	1.020 (0.963–1.090)
CLEC4A	0.790	1.020 (0.884–1.180)	GSTT2	0.426	0.957 (0.860–1.070)
DNALI1	0.788	0.988 (0.907–1.080)	NCF1	0.228	1.060 (0.962–1.180)
COQ4	0.966	0.997 (0.887–1.120)	S100A9	0.530	1.030 (0.944–1.120)
PLK2	0.363	0.947 (0.843–1.060)	CD72	0.876	1.010 (0.904–1.130)
CUEDC1	0.853	1.010 (0.911–1.120)	TSPAN1	0.603	1.030 (0.933–1.130)
GZMB	0.627	1.040 (0.900–1.190)	KCNK15	0.800	1.010 (0.916–1.120)
GUSBP3	0.448	1.050 (0.926–1.190)	IGF2BP2	0.918	1.010 (0.908–1.110)
ST3GAL1	0.940	1.000 (0.907–1.110)	IGFBP2	0.219	1.070 (0.960–1.200)
GFRA1	0.165	1.080 (0.970–1.190)	FCGR3B	0.277	1.060 (0.958–1.160)
BCL2A1	0.890	0.992 (0.887–1.110)	SCGB2A2	0.064	1.050 (0.997–1.110)
S100A8	0.254	1.050 (0.969–1.130)	MAOB	0.744	0.987 (0.910–1.070)
CLSTN2	0.383	1.050 (0.938–1.180)	ADIRF	0.987	0.999 (0.929–1.080)
GSTZ1	0.987	0.999 (0.903–1.110)	PDZK1	0.459	1.040 (0.941–1.140)
NAT1	0.263	1.050 (0.965–1.140)	WIF1	0.296	0.951 (0.865–1.050)
LAMA3	0.927	0.995 (0.897–1.100)	DUSP4	0.935	1.000 (0.913–1.100)
FCGR3A	0.174	1.080 (0.966–1.210)	CNN1	0.878	0.992 (0.893–1.100)
ATP13A2	0.200	0.928 (0.828–1.040)	NPY1R	0.987	0.999 (0.938–1.060)
EPN3	0.323	1.060 (0.941–1.200)	KRT14	0.780	1.010 (0.947–1.070)
PEG10	0.206	0.941 (0.856–1.030)	VTCN1	0.123	0.926 (0.840–1.020)
CA12	0.227	1.070 (0.957–1.210)	MNDA	0.483	1.030 (0.942–1.140)
ZNF580	0.369	0.944 (0.831–1.070)	SERHL	0.841	0.991 (0.905–1.080)
TBX3	0.260	0.951 (0.871–1.040)	SLC44A4	0.128	1.090 (0.977–1.210)
C7orf63	0.087	1.120 (0.984–1.280)	AQP9	0.916	1.000 (0.924–1.090)
ARNT2	0.587	1.030 (0.922–1.150)	CXCL8	0.444	0.962 (0.871–1.060)
MS4A1	0.306	1.070 (0.938–1.230)	MGP	0.442	1.040 (0.941–1.150)
MAPT	0.766	0.981 (0.863–1.110)	SGK3	0.891	0.993 (0.901–1.090)
SELL	0.167	1.090 (0.966–1.220)	NKG7	0.579	0.980 (0.914–1.050)
CCL5	0.345	1.060 (0.939–1.200)	CTGF	0.657	0.979 (0.894–1.070)
BAMBI	0.098	0.897 (0.789–1.020)	DHRS2	0.742	0.986 (0.909–1.070)

*Note:* The multiple boldfaced data in Table 1 denote significance, as mentioned in Section 3.2.

### 3.3. Functional Analysis

The functions of these five drug‐resistant DEGs related to survival were further analyzed. DACH1 is involved in transcriptional regulation, GREB1 plays an important role in the estrogen signaling pathway, STAP1 is involved in signal transduction, and TDRD12 is involved in cell division and other processes, as shown in Table 2.

**Table 2:** Functional analysis.

ID	Gene name	GOTERM_BP_DIRECT	KEGG_PATHWAY
DACH1	Dachshund family transcription Factor 1	GO:0000122 ~ negative regulation of transcription by RNA Polymerase II,	—
		GO:0001967 ~ suckling behavior,	
		GO:0006357 ~ regulation of transcription by RNA Polymerase II,	
		GO:0007585 ~ respiratory gaseous exchange by respiratory system,	
		GO:0010944 ~ negative regulation of transcription by competitive promoter binding,	
		GO:0030336 ~ negative regulation of cell migration,	
		GO:0033262 ~ regulation of nuclear cell cycle DNA replication,	
		GO:0044342 ~ Type B pancreatic cell proliferation,	
		GO:0045892 ~ negative regulation of DNA‐templated transcription,	
		GO:0046545 ~ development of primary female sexual characteristics,	
		GO:0048147 ~ negative regulation of fibroblast proliferation,	
		GO:0060244 ~ negative regulation of cell proliferation involved in contact inhibition,	
		GO:2000279 ~ negative regulation of DNA biosynthetic process,	


GREB1	Growth regulating estrogen receptor Binding 1	GO:0002009 ~ morphogenesis of an epithelium,	hsa04915:estrogen signaling pathway,

SCGB1D2	Secretoglobin Family 1D Member 2	—	—

STAP1	Signal transducing adaptor family Member 1	GO:0007169 ~ cell surface receptor protein tyrosine kinase signaling pathway,	—
		GO:0010628 ~ positive regulation of gene expression,	
		GO:0010760 ~ negative regulation of macrophage chemotaxis,	
		GO:0042326 ~ negative regulation of phosphorylation,	
		GO:0050861 ~ positive regulation of B cell receptor signaling pathway,	
		GO:0060100 ~ positive regulation of phagocytosis, engulfment,	
		GO:0071222 ~ cellular response to lipopolysaccharide,	
		GO:1900028 ~ negative regulation of ruffle assembly,	
		GO:1902227 ~ negative regulation of macrophage colony‐stimulating factor signaling pathway,	
		GO:1903980 ~ positive regulation of microglial cell activation,	
		GO:1904140 ~ negative regulation of microglial cell migration,	
		GO:1904151 ~ positive regulation of microglial cell mediated cytotoxicity,	

TDRD12	Tudor domain containing 12	GO:0007140 ~ male meiotic nuclear division,	—
		GO:0007283 ~ spermatogenesis,	
		GO:0009566 ~ fertilization,	
		GO:0030154 ~ cell differentiation,	
		GO:0031047 ~ regulatory ncRNA‐mediated gene silencing,	
		GO:0034587 ~ piRNA processing,	
		GO:0042078 ~ germ‐line stem cell division,	
		GO:0051321 ~ meiotic cell cycle,	
		GO:0141196 ~ transposable element silencing by piRNA‐mediated DNA methylation,	

### 3.4. Immune Correlation Analysis

**Figure 3:**
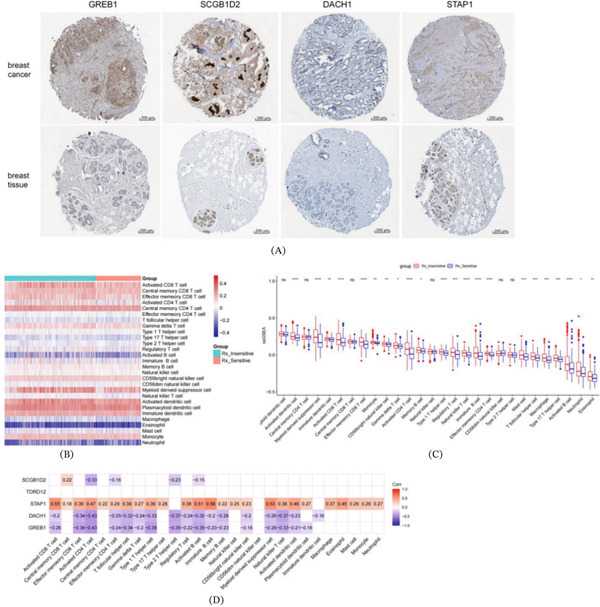
Immune correlation analysis. (A) Comparison of immunohistochemical images between normal tissues and tumor tissues; (B) heatmap of immune infiltration levels; (C) comparison of immune cell infiltration levels between the Rx_Insensitive group and the Rx_Sensitive group; (D) correlation analysis of expression levels of GREB1, DACH1, STAP1, TDRD12, and SCGB1D2 with immune cell infiltration levels.

By comparing immunohistochemical images between normal tissues and tumor tissues through the The Human Protein Atlas database, GREB1, SCGB1D2, and STAP1 were highly expressed in breast tumor tissues, while DACH1 was lowly expressed in breast tumor tissues, as shown in Figure 3A. The immune infiltration levels of each patient were quantified through expression profiling (see Figure 3B). The immune cell infiltration conditions of the Rx_Insensitive group and the Rx_Sensitive group were compared. The infiltration levels of most immune cells in the Rx_Insensitive group were higher than those in the Rx_Sensitive group (Figure 3C). The expression levels of GREB1, SCGB1D2, DACH1, and STAP1 were significantly correlated with the infiltration levels of various immune cells (*p* < 0.05, Figure 3D).

### 3.5. Construct a Prognostic Model and Model Evaluation

**Figure 4:**
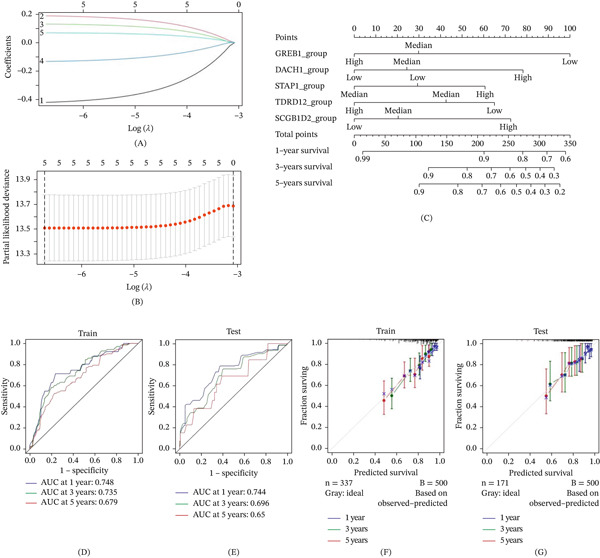
Construction of Prognostic Model. (A) LASSO path diagram, each curve represents the trajectory of the change in each independent variable coefficient. The vertical axis represents the value of the coefficient, the lower horizontal axis represents log (*λ*), and the upper horizontal axis represents the number of nonzero coefficients in the model at this time. It can be seen that as the parameter log *λ* increases, the regression coefficient (i.e., the value on the vertical axis) continuously converges and eventually converges to 0.; (B) 10‐fold cross‐validation to select the most appropriate clinical features, The left dotted line represents *λ* min, which indicates the *λ* value when the deviation is the smallest, indicating that the model fitting effect is the best under this *λ* value. The number of variables is 5, and compared with *λ* ‐se, more variables are retained. The right dotted line is *λ* ‐se, meaning one standard error on the right side of the minimum *λ*. Under this *λ* value, the number of variables included in the equation is less (0). Therefore, the left *λ* min is selected as the final equation selection criterion; (C) Construction of a prognostic nomogram model for breast cancer patients; (D)The ROC curve for 1, 3, and 5 years were used to evaluate the predictive efficacy of the training set; (E) The ROC curve for 1, 3, and 5 years were used to evaluate the predictive efficacy of the validation set; (F) The calibration curve for 1, 3, and 5 years were used to evaluate the predictive efficacy of the training set; (G) The calibration curve for 1, 3, and 5 years were used to evaluate the predictive efficacy of the validation set.

Given that these five genes (GREB1, DACH1, STAP1, TDRD12, and SCGB1D2) showed significant differences in drug resistance, survival outcomes, and immune‐related analyses, a LASSO‐Cox regression model was further constructed to evaluate their prognostic robustness and reduce potential overfitting in the model. The optimal penalty parameter (*λ*) was determined by 10‐fold cross‐validation using the minimum mean cross‐validated partial likelihood deviance. As shown in Figure 4A, the cross‐validation curve identified the optimal *λ* value corresponding to the simplest model with minimal prediction error. At the selected *λ* value, all five genes retained nonzero coefficients in the LASSO model (Figure 4B), indicating that each gene contributed to the prognostic signature. The coefficient profiles demonstrated stable shrinkage without elimination of variables across the *λ* sequence, supporting the robustness of these candidate genes as predictors. Therefore, this study performed prognostic modeling based on these five genes (Figure 4C). For example, if a patient has low expression of GREB1, high expression of DACH1, high expression of STAP1, low expression of TDRD12, and low expression of SCGB1D2, then the corresponding risk score is 100 + 78 + 60 + 65 + 0 = 303. The 1‐year DRFS survival rate is approximately 70%, the 3‐year DRFS survival rate is approximately 40%, and the 5‐year DRFS survival rate is approximately 30%.

To evaluate the predictive value of the model, we randomly selected 2/3 of the samples (337 cases) for modeling and 1/3 of the samples (171 cases) for validation. Through ROC curve analysis, the 1‐year AUC of this model was 0.748, the 3‐year AUC was 0.735, and the 5‐year AUC was 0.679 (see Figure 4D). In the validation set, the predicted 1‐year AUC was 0.744, the 3‐year AUC was 0.696, and the 5‐year AUC was 0.650 (Figure 4E). The calibration curve shows that the predicted probabilities of the model are close to the true values (Figure 4F,G).

### 3.6. External Model Validation

**Figure 5:**
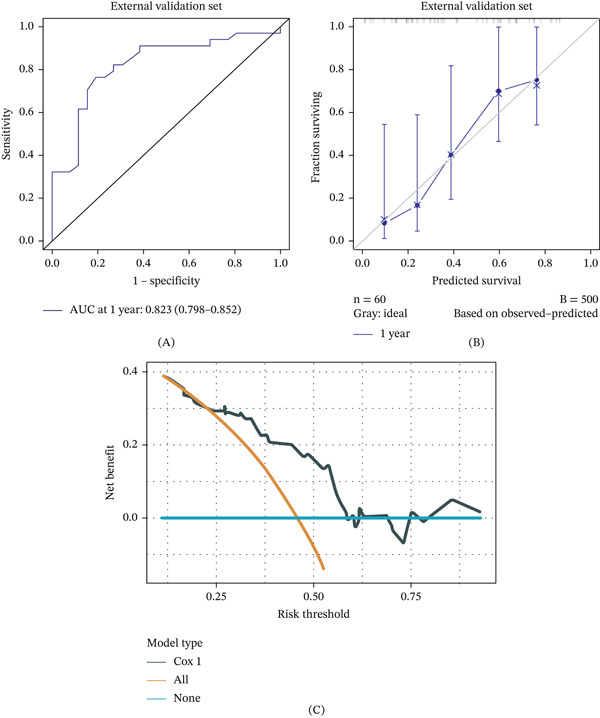
External model validation. (A) The ROC curve for 1 year was used to evaluate the predictive efficacy of the external validation set; (B) The calibration curve for 1 year was used to evaluate the predictive efficacy of the external validation set; (C) The decision curve for 1 year was used to evaluate the predictive efficacy of the external validation set. The decision curve is above the two extreme curves, indicating that the model is valuable (the orange line represents the assumption that all patients have the disease, the blue line represents the assumption that no patients have the disease, and the black line represents the decision curve of the model).

A total of 60 clinical samples were collected, and follow‐up was conducted for the patients. The median follow‐up time was 0.839 years (307 days). During the follow‐up period, 37 cases experienced DRFS events. The clinical characteristics of the patients are shown in Table 3. The nomogram model was used to evaluate the DRFS survival rate of the 60 patients. In the external validation cohort, the prognostic model demonstrated good predictive performance for 1‐year survival. The concordance index (C‐index) was 0.823 (95% CI: 0.798–0.852), indicating favorable discrimination ability. The time‐dependent ROC analysis further showed that the area under the curve (AUC) for 1‐year survival was 0.823. (Figure 5A). The calibration curve showed that the predicted probabilities of the model were close to the true values (Figure 5B). The curve in the decision curve indicates that the model′s predictions are helpful for decision‐making when the probability threshold is between 0.25 and 0.6 (Figure 5C). Although long‐term validation remains limited, the current results still demonstrate good short‐term predictive performance and clinical utility.

**Table 3:** Clinical characteristics of 60 patients.

Characteristic	*D* *R* *F* *S* = 1	*D* *R* *F* *S* = 0	*χ* ^2^/t/Z	*p*
Age	50.04 ± 12.04	49.54 ± 10.75	0.162	0.872
ER status (N/P)	29/8	12/11	4.501	0.034
PR status (N/P)	30/7	17/6	0.429	0.512
HER2 status (N/P)	36/1	22/1	0.119	0.730
ERBB2 status (N/P)	34/3	23/0	1.963	0.161
Clinical AJCC stage (IIA/IIB/IIIA/IIIB/IIIC)	1/9/12/12/3	5/4/7/7/0	7.355	0.118
Grade (1/2/3)	1/9/27	1/11/11	3.882	0.144
DRFS event time (years)	1.032 [0.854, 1.128]	0.728 [0.458, 0.854]	−4.465	< 0.001

## 4. Discussion

Chemotherapy is a key treatment method for breast cancer. However, the emergence of drug resistance has become one of the main obstacles leading to treatment failure. Studies have revealed that the expression levels of certain genes related to chemotherapy resistance in breast cancer patients are closely linked to the prognosis of the disease [9]. By precisely identifying these drug resistance‐related genes and constructing corresponding prognostic models, patients can be accurately stratified into high‐risk and low‐risk groups based on the expression levels of these genes. These two groups show significant differences in prognosis and immune responses [10]. Moreover, there are significant differences in the response of different patients to chemotherapy drugs, and part of the reason can be attributed to individual differences in gene expression. Therefore, precisely identifying drug resistance‐related genes and tailoring personalized treatment plans for patients is of crucial significance for improving treatment outcomes. Based on this, this study—relying on the large‐scale sample dataset in the GEO database—successfully identified five key genes closely related to the response of breast cancer to neoadjuvant taxane‐anthracycline chemotherapy. They are GREB1, DACH1, STAP1, TDRD12, and SCGB1D2. In particular, GREB1, DACH1, STAP1, and SCGB1D2 are significantly correlated with the infiltration levels of various immune cells.

The expression level of the GREB1 (Growth Regulation by Estrogen in Breast Cancer 1) gene in breast cancer tissues is higher than that in normal breast tissues. In breast cancer, Mohammed et al. [11] found that the level of GREB1 is a good prognostic factor independent of the Nottingham Prognostic Index (NPI). The NPI predicts the postoperative prognosis of breast cancer based on the size of the lesion, the number of involved lymph nodes, and the tumor grade. In the analysis of 419 breast cancer patients, GREB1‐positive (Allred > 2) estrogen receptor‐positive (ER+) breast cancer patients with positive results had better clinical outcomes, with an HR of 0.49 (0.26–0.90), *p* = 0.021 [11]. The KM analysis results of this study also indicated that the patients in the high‐expression group of GREB1 had significantly better disease‐free recurrence‐free survival (DRFS) than those in the low‐expression group, suggesting that high expression has better prognostic results. The multivariate COX regression analysis showed that GREB1 (HR = 0.653 [0.532–0.802)], *p* < 0.05.

DACH1 (Dachshund homolog 1) is an important transcription factor. It plays a crucial role in the development and differentiation of cells. DACH1 interacts with *β*‐catenin to regulate the activity of the Wnt signaling pathway. Additionally, DACH1 is involved in the regulation of the Notch signaling pathway, influencing cell differentiation and fate determination. DACH1 is expressed in normal human breast epithelial cells, but its protein level is downregulated in many cases of breast cancer, which is significantly associated with poor prognosis [12]. An analysis of 3574 breast cancer patients from 19 public databases (GEO) showed that high expression of DACH1 mRNA was associated with longer overall survival (OS), recurrence‐free survival (RFS), and metastasis‐free survival (MFS) [13]. However, this study found that high expression of DACH1 mRNA was associated with lower DFS. Possible reasons for this could include DACH1 regulating the expression of certain cell cycle proteins, influencing the cell cycle process, and thereby promoting tumor cell proliferation under specific conditions. In triple‐negative breast cancer (TNBC), DACH1 expression is usually low, which may be associated with a poorer prognosis. Therefore, high expression of DACH1 may mask its role as a tumor suppressor gene in certain situations. The expression level of DACH1 is influenced by epigenetic regulation and miRNA regulation, and the tumor heterogeneity of breast cancer may lead to differences in the prognostic value of DACH1 in different studies.

The STAP family, including STAP‐1 and STAP‐2, are involved in various intracellular signaling pathways. During tumor development and inflammatory/immune responses, STAP proteins interact with kappaB kinase inhibitors, breast tumor kinase, signal transducer and activator of transcription 3 (STAT3), and STAT5. STAP proteins positively or negatively regulate key steps in intracellular signaling pathways through their unique mechanisms [14]. Previous studies have shown that the median survival time, OS, and DFS of patients with high methylation expression of STAP1 are all shorter than those of patients with low methylation expression of hepatocellular carcinoma [15]. The results of this study also indicated that patients with high STAP1 expression tended to have shorter disease recurrence‐free survival (DRFS) (HR = 1.140, 95% CI = 1.016–1.279).

The protein encoded by the TDRD12 gene belongs to the Tudor domain family. Proteins in this family usually participate in the binding and regulation of RNA and DNA. Studies have shown that the expression level of TDRD proteins in breast cancer is usually lower than that in normal breast tissue. The low expression of TDRD proteins is associated with the malignant phenotype and poor prognosis of breast cancer [16]. The results of this study also show that the survival rate of patients with low expression of TDRD12 is lower than that of patients with high expression in DRFS.

SCGB1D2 (Secretoglobin Family 1D Member 2), also known as Lipophilin B, is a member of the Secretoglobins family. The genes of this family are usually related to the production of secretory proteins, which play various roles in the human body, from immune response to cell communication. Previous studies have shown that the expression level of Lipophilin B in breast cancer is usually higher than that in normal breast tissue [17]. However, its impact on the prognosis of breast cancer has not been reported in previous studies. The results of this study show that the elevated expression level of SCGB1D2 in breast cancer patients is closely related to the poor prognosis, with HR = 1.074, *p* < 0.05.

GO and KEGG enrichment analyses in this study revealed that the identified genes were mainly involved in biological processes related to cell cycle regulation, transcriptional and epigenetic control, epithelial–mesenchymal transition (EMT), and tumor immune microenvironment modulation. These pathways have been widely reported to play critical roles in the development of chemoresistance and unfavorable prognosis in breast cancer [18, 19]. Dysregulation of cell cycle progression and DNA replication is closely associated with enhanced proliferative capacity and reduced sensitivity to DNA‐damaging chemotherapeutic agents. In parallel, transcriptional reprogramming and noncoding RNA–mediated gene silencing contribute to the activation of drug efflux transporters and survival pathways, thereby facilitating acquired resistance. Moreover, EMT‐related processes and epithelial morphogenesis alterations are recognized as key drivers of tumor cell plasticity, metastasis, and therapy resistance. In addition, enrichment of immune‐related pathways suggests a potential involvement of tumor microenvironment remodeling, particularly macrophage‐ and lymphocyte‐associated signaling, which has been shown to influence both chemotherapy response and long‐term patient survival. Collectively, these findings support the notion that the identified gene set may contribute to breast cancer progression and chemoresistance through coordinated regulation of proliferation, transcriptional control, cellular plasticity, and immune modulation.

In breast cancer patients undergoing NAC with paclitaxel and anthracycline drugs, the expression levels of the five genes, namely, GREB1, DACH1, STAP1, TDRD12, and SCGB1D2, may be closely related to the DRFS of the patients, and they are expected to become biomarkers for predicting the prognosis of the patients. Based on these five key genes, we constructed a new breast cancer DRFS prognosis model and conducted a comprehensive evaluation of it. The results showed that this newly developed prognosis model demonstrated good predictive performance in predicting the 1‐, 3‐, and 5‐year DRFS survival periods of the patients, with the AUC values of 0.748, 0.735, and 0.679, respectively, for 1, 3, and 5 years. Moreover, the calibration curve results also further indicated that the model was closer to the actual results and could provide a relatively accurate prognosis assessment reference for clinical practice. In the field of breast cancer prognosis models, numerous previous studies have achieved remarkable results. For instance, Zhang et al. [20] constructed an independent prognostic factor model consisting of 11 Tumor‐Associated Neutrophils (TANs) related genes. They predicted the total survival periods of 3, 5, and 7 years in the TCGA (The Cancer Genome Atlas) training cohort and calculated the AUC values as 0.72, 0.73, and 0.73, respectively. Another example is Huang et al. [21], who focused on exosome‐related genes and established a prognostic model consisting of three genes (JUP, CAPZA1, and ARVCF). The AUC values of this model in predicting the survival periods of patients for 1, 3, and 5 years were 0.654, 0.602, and 0.635, respectively. Lastly, Yu et al. [22] developed a risk model based on the prognostic genes related to breast cancer cytochrome c (Cyt c), consisting of eight genes related to Cyt c. The AUC values of this model at 1, 3, and 5 years were 0.80, 0.77, and 0.74, respectively, demonstrating excellent predictive performance. However, compared with these previous studies, the breast cancer DRFS prognostic model constructed in this study, which is based on five chemotherapy response‐related genes, although its AUC values at certain time points may be slightly lower than those of some previous studies′ models, still has unique advantages and value. Firstly, this model focuses on the specific clinical treatment context of neoadjuvant taxane‐anthracycline chemotherapy, being more targeted and able to provide more precise prognostic assessment for breast cancer patients receiving such chemotherapy regimens. This was verified in the clinical samples. Specifically, this study collected 60 samples for external validation. The results showed that AUC = 0.823, indicating that the model has a moderately high predictive performance. Secondly, the construction of this model is based on in‐depth analysis of the expression levels of these five key genes, providing a new perspective and basis for breast cancer prognosis assessment at the genetic level. Moreover, the calibration curve results of this model also indicate its certain predictive value, which can provide beneficial references for clinicians in formulating treatment plans and managing patients′ prognosis, and help improve the scientificity and accuracy of clinical decisions.

Nevertheless, the training dataset (GSE25066) was generated over a decade ago and that this may introduce potential limitations in terms of population heterogeneity and evolving clinical practice. Although the model shows promising predictive performance, prospective validation in contemporary multicenter cohorts is required before clinical application. The external validation cohort is derived from a single‐center real‐world dataset with a relatively small sample size, which may limit the generalizability of the findings. In the future, we will further optimize this model, expand the sample size for verification, and explore its application potential in different clinical scenarios, with the aim of providing more powerful support for the precise prognosis assessment and individualized treatment of breast cancer patients. The current findings are based on computational analyses and correlation, and that causal relationships and underlying molecular mechanisms remain to be further investigated. Future research directions should include conducting in vitro and in vivo functional experiments (such as gene knockdown/overexpression experiments, cell viability and drug sensitivity assays) as well as animal model studies to verify the mechanism of these genes in chemotherapy resistance.

## 5. Conclusion

Based on publicly available GEO data, this study identified five prognostic genes related to chemotherapy response in breast cancer: GREB1, DACH1, STAP1, TDRD12, and SCGB1D2. Using these five genes, a nomogram model was effectively constructed, and this model demonstrated good predictive performance in terms of DFS period.

## Funding

No funding was received for this manuscript.

## Disclosure

All the authors agreed to publish the article.

## Conflicts of Interest

The authors declare no conflicts of interest.

## Data Availability

The original contributions presented in this study are included in the article. Further inquiries can be directed to the corresponding author.

## References

[1] Chien A. J. , Tripathy D. , Albain K. S. , Symmans W. F. , Rugo H. S. , Melisko M. E. , Wallace A. M. , Schwab R. , Helsten T. , Forero-Torres A. , Stringer-Reasor E. , Ellis E. D. , Kaplan H. G. , Nanda R. , Jaskowiak N. , Murthy R. , Godellas C. , Boughey J. C. , Elias A. D. , Haley B. B. , Kemmer K. , Isaacs C. , Clark A. S. , Lang J. E. , Lu J. , Korde L. , Edmiston K. K. , Northfelt D. W. , Viscusi R. K. , Yee D. , Perlmutter J. , Hylton N. M. , Van′t Veer L. J. , DeMichele A. , Wilson A. , Peterson G. , Buxton M. B. , Paoloni M. , Clennell J. , Berry S. , Matthews J. B. , Steeg K. , Singhrao R. , Hirst G. L. , Sanil A. , Yau C. , Asare S. M. , Berry D. A. , and Esserman L. J. , I-SPY 2 Consortium. MK-2206 and Standard Neoadjuvant Chemotherapy Improves Response in Patients With Human Epidermal Growth Factor Receptor 2-Positive and/or Hormone Receptor-Negative Breast Cancers in the I-SPY 2 Trial, Journal of Clinical Oncology. (2020) 38, no. 10, 1059–1069, 10.1200/JCO.19.01027. 32031889 PMC7106976

[2] Rastogi P. , Anderson S. J. , Bear H. D. , Geyer C. E. , Kahlenberg M. S. , Robidoux A. , Margolese R. G. , Hoehn J. L. , Vogel V. G. , Dakhil S. R. , Tamkus D. , King K. M. , Pajon E. R. , Wright M. J. , Robert J. , Paik S. , Mamounas E. P. , and Wolmark N. , Preoperative Chemotherapy: Updates of National Surgical Adjuvant Breast and Bowel Project Protocols B-18 and B-27, Journal of Clinical Oncology. (2008) 26, no. 5, 778–785, 10.1200/JCO.2007.15.0235. 18258986

[3] Chen X. , Ye G. , Zhang C. , Li X. , Chen Y. , Xie X. , Zheng H. , Cao Y. , Wu K. , Ni D. , Tang J. , Wei Z. , and Shen K. , Superior Outcome After Neoadjuvant Chemotherapy With Docetaxel, Anthracycline, and Cyclophosphamide Versus Docetaxel Plus Cyclophosphamide: Results From the NATT Trial in Triple Negative or HER2 Positive Breast Cancer, Breast Cancer Research and Treatment. (2013) 142, no. 3, 549–558, 10.1007/s10549-013-2761-1. 24292815

[4] Senkus E. , Kyriakides S. , Ohno S. , Penault-Llorca F. , Poortmans P. , Rutgers E. , Zackrisson S. , and Cardoso F. , ESMO Guidelines Committee. Primary Breast Cancer: ESMO Clinical Practice Guidelines for Diagnosis, Treatment and Follow-Up, Annals of Oncology. (2015) 26, no. suppl ement 5, v8–30, 10.1093/annonc/mdv298. 26314782

[5] Pivot X. , Asmar L. , Buzdar A. U. , Valero V. , and Hortobagyi G. , A Unified Definition of Clinical Anthracycline Resistance Breast Cancer, British Journal of Cancer. (2000) 82, no. 3, 529–534, 10.1054/bjoc.1999.0958. 10682660 PMC2363337

[6] Grimsley H. E. , Antczak M. , Reddin I. G. , Weiler N. , McLaughlin K. M. , Rothweiler F. , Haas J. , Nist A. , Mernberger M. , Stiewe T. , Fenton T. R. , Speidel D. , Harper-Wynne C. , Cox K. , Heckl D. , Cinatl J. , Wass M. N. , Garrett M. D. , and Michaelis M. , Using a Novel Panel of Drug-Resistant Triple-Negative Breast Cancer Cell Lines to Identify Candidate Therapeutic Targets and Biomarkers, Cancer Letters. (2025) 1, no. 624, 217754, 10.1016/j.canlet.2025.217754. 40300663

[7] Qiu P. , Yu X. , Zheng F. , Gu X. , Huang Q. , Qin K. , Hu Y. , Liu B. , Xu T. , Zhang T. , Chen G. , and Liu Y. , Advancements in Liquid Biopsy for Breast Cancer: Molecular Biomarkers and Clinical Applications, Cancer Treatment Reviews. (2025) 139, 102979, 10.1016/j.ctrv.2025.102979. 40540857

[8] Hatzis C. , Pusztai L. , Valero V. , Booser D. J. , Esserman L. , Lluch A. , Vidaurre T. , Holmes F. , Souchon E. , Wang H. , Martin M. , Cotrina J. , Gomez H. , Hubbard R. , Chacón J. I. , Ferrer-Lozano J. , Dyer R. , Buxton M. , Gong Y. , Wu Y. , Ibrahim N. , Andreopoulou E. , Ueno N. T. , Hunt K. , Yang W. , Nazario A. , DeMichele A. , O′Shaughnessy J. , Hortobagyi G. N. , and Symmans W. F. , A Genomic Predictor of Response and Survival Following Taxane-Anthracycline Chemotherapy for Invasive Breast Cancer, JAMA. (2011) 305, no. 18, 1873–1881, 10.1001/jama.2011.593. 21558518 PMC5638042

[9] Sarkar T. , Sarkar T. , Goswami M. , Robaszkiewicz A. , and Sarkar K. , Integrated Transcriptome Analysis and In Silico Investigations Identify KHDRBS3 to Target With Steroidal Lactone in Paclitaxel Resistance Breast Cancer, 152332Biochemical and Biophysical Research Communications. (2025) 777, 10.1016/j.bbrc.2025.152332, 40651363. 40651363

[10] Liu Y. , Dong L. , Ma J. , Chen L. , Fang L. , and Wang Z. , The Prognostic Genes Model of Breast Cancer Drug Resistance Based on Single-Cell Sequencing Analysis and Transcriptome Analysis, Clinical and Experimental Medicine. (2024) 24, no. 1, 10.1007/s10238-024-01372-6. PMC11127859 38795164

[11] Mohammed H. , D′Santos C. , Serandour A. A. , Ali H. R. , Brown G. D. , Atkins A. , Rueda O. M. , Holmes K. A. , Theodorou V. , Robinson J. L. , Zwart W. , Saadi A. , Ross-Innes C. S. , Chin S. F. , Menon S. , Stingl J. , Palmieri C. , Caldas C. , and Carroll J. S. , Endogenous Purification Reveals GREB1 as a Key Estrogen Receptor Regulatory Factor, Cell Reports. (2013) 3, no. 2, 342–349, 10.1016/j.celrep.2013.01.010. 23403292 PMC7116645

[12] Wu K. , Liu M. , Li A. , Donninger H. , Rao M. , Jiao X. , Lisanti M. P. , Cvekl A. , Birrer M. , and Pestell R. G. , Cell Fate Determination Factor DACH1 Inhibits C-Jun-Induced Contact-Independent Growth, Molecular Biology of The Cell. (2007) 18, no. 3, 755–767, 10.1091/mbc.e06-09-0793. 17182846 PMC1805093

[13] Xu H. , Yu S. , Yuan X. , Xiong J. , Kuang D. , Pestell R. G. , and Wu K. , DACH1 Suppresses Breast Cancer as a Negative Regulator of CD44, Scientific Reports. (2017) 7, no. 1, 10.1038/s41598-017-04709-2. PMC5489534 28659634

[14] Matsuda T. and Oritani K. , Possible Therapeutic Applications of Targeting STAP Proteins in Cancer, Biological and Pharmaceutical Bulletin. (2021) 44, no. 12, 1810–1818, 10.1248/bpb.b21-00672. 34853263

[15] Sun L. , Zhao X. , Zhang H. , Li G. , and Li N. , Relationship Between STAP1 Methylation in Peripheral Blood T Cells and the Clinicopathological Characteristics and Prognosis of Patients Within 5-Cm Diameter HCC, Minerva Gastroenterology. (2024) 70, no. 1, 16–21, 10.23736/S2724-5985.23.03309-0. 37526444

[16] Jiang Y. , Liu L. , Shan W. , and Yang Z. Q. , An Integrated Genomic Analysis of Tudor Domain-Containing Proteins Identifies PHD Finger Protein 20-Like 1 (PHF20L1) as a Candidate Oncogene in Breast Cancer, Molecular Oncology. (2016) 10, no. 2, 292–302, 10.1016/j.molonc.2015.10.013. 26588862 PMC4740244

[17] Culleton J. , O′Brien N. , Ryan B. M. , Hill A. D. , McDermott E. , O′Higgins N. , and Duffy M. J. , Lipophilin B: A Gene Preferentially Expressed in Breast Tissue and Upregulated in Breast Cancer, International Journal of Cancer. (2007) 120, no. 5, 1087–1092, 10.1002/ijc.22471. 17163411

[18] Vuoti S. , Saari M. , Lahti J. , Narasimha K. , and Reinikainen K. , Association of Baseline Tumor-Infiltrating Lymphocytes and Cell-Cycle Regulation Markers on Prognosis and Mortality in Patients With Advanced Breast Cancer According to Tumor Characteristics and Treatment Type: An Observational Study, Breast Cancer Research and Treatment. (2026) 217, no. 2, 10.1007/s10549-026-07991-9. PMC13236729 42243565

[19] Chen C. , Han C. , Xue L. , and Lu X. , Exercise-Related Genes Predicts Overall Survival and Tumor Immune Microenvironment, and Identifies the Biological Role of SLC52A2 in Breast Cancer, Discover Oncology, 2026, SpringerNature, 10.5772/intechopen.1008585. PMC13424061 42215809

[20] Zhang J. , Wang X. , Zhang Z. , Ma F. , and Wang F. , A Novel Tumor-Associated Neutrophil Gene Signature for Predicting Prognosis, Tumor Immune Microenvironment, and Therapeutic Response in Breast Cancer, Scientific Reports. (2024) 14, no. 1, 10.1038/s41598-024-55513-8. PMC10912776 38438469

[21] Huang G. , Yu Y. , Su H. , Gan H. , and Chu L. , Integrating RNA-Seq and scRNA-Seq to Explore the Prognostic Features and Immune Landscape of Exosome-Related Genes in Breast Cancer Metastasis, Annals of Medicine. (2025) 57, no. 1, 2447917, 10.1080/07853890.2024.2447917. 39847423 PMC11758802

[22] Yu H. , Li S. , Wu J. , and Wang H. , Identification and Experimental Validation of Prognostic Genes Related to Cytochrome C in Breast Cancer, Frontiers in Genetics. (2025) 11, no. 16, 10.3389/fgene.2025.1627134. PMC12375475 40860340

